# Establishment and functional characterization of a murine primary Sertoli cell line deficient of connexin43

**DOI:** 10.1007/s00441-020-03203-y

**Published:** 2020-04-23

**Authors:** Jonathan Gerber, Kristina Rode, Nina Hambruch, Marion Langeheine, Nadine Schnepel, Ralph Brehm

**Affiliations:** Institute of Anatomy, University of Veterinary Medicine Hannover Foundation, Hannover, Germany

**Keywords:** Blood-testis barrier, Cell culture model, Claudin-11, Gap junctions, SCCx43KO

## Abstract

The Sertoli cell (SC) specific connexin43 (Cx43) knockout (SCCx43KO) mouse line is ideal to gain insight into the mechanistic gap junction formation in SC and the seminiferous epithelium. A method for developing primary SC cultures from these mice was established, validated and successfully characterized via polymerase chain reaction, immunohistochemistry, immunofluorescence (IF), and Western blots (WB). It was evident that both knockout (KO) and wild-type (WT) primary cell cultures were similar in morphology. These highly pure SC cultures were subjected to cell proliferation assays indicating no notable proliferation in cultures of both genotypes. Measurements of cell monolayer integrity indicated significant increases in transepithelial electrical resistance and consequently in tight junction expression of the KO cultures. Using semi-quantitative WB and IF, tight junction protein claudin-11 was analyzed. These results support a role for Cx43 in regulating blood-testis barrier (BTB) function, composition, and dynamics in vitro. Thus, the SC deficient Cx43 cell cultures may provide a valuable in vitro tool for a better understanding of the mechanistic role of Cx43 in spermatogenesis and BTB assembly.

**Electronic supplementary material:**

The online version of this article (10.1007/s00441-020-03203-y) contains supplementary material, which is available to authorized users.

## Introduction

A gap junction channel is composed of two hemichannels called connexons, which are responsible for direct intercellular communication between adjoining cells. Each cell contains one connexon, composed of six connexin (Cx) proteins (Kumar and Gilula 1996; Maeda et al. 2009; Tripathi and Tripathi 2010). Gap junctions originally have been found to be involved in the transport of small molecules and ions (< 1 kDa) between connected cells (Kumar and Gilula 1996; Loewenstein 1981). Over the past couple of years, other functions have been linked to gap junctions: a dynamic cell modulation of the cytoskeletal structure as well as extrinsic guidance to promote cell-cell adhesion (Giepmans et al. 2001; Giese et al. 2012; Hartsock and Nelson 2008; Huang et al. 1998; Itoh et al. 1997; Kameritsch et al. 2012; Stout et al. 2004). Single hemichannels might also regulate physiological roles by controlling adenosine triphosphate (ATP), nicotinamide adenine dinucleotide (NAD^+^), and Ca^2+^ wave signaling (Pointis et al. 2010; Spray et al. 2006; Stout et al. 2004), and these junctions tend to be selective, aiding in cellular growth and differentiation (Bruzzone et al. 1996; Kumar and Gilula 1996; Warner et al. 1984). There are at least twenty different Cx genes coding for gap junction proteins in mice (Sohl and Willecke 2004).

The gap junction gene *Gja1* (also known as gap junction protein, alpha 1) codes for one of the most researched gap junction protein known as Cx43. In the seminiferous epithelium, gap junctional Cx43 is located in the cell membrane of adjacent Sertoli cells (SC) and between SC and germ cells (GC), where it is involved in testicular development, GC and SC differentiation and spermatogenesis (Bravo-Moreno et al. 2001; Decrouy et al. 2004; Gerber et al. 2014; Gunther et al. 2013). SC nurture the developing GC and aid in their development and translocation from the basal to the adluminal compartment of the seminiferous tubule (Brehm et al. 2007; Cheng and Mruk 2012; Gerber et al. 2014; Pointis and Segretain 2005; Sridharan et al. 2007; Tripathi and Tripathi 2010). In particular, some men who are diagnosed with testicular carcinoma in situ (CIS) exhibit a downregulation of Cx43 between SC, SC-GC, and tumor cells (Brehm et al. 2002; Brehm et al. 2006). Additionally, male factor infertility due to impaired spermatogenesis might also be caused in some men by a downregulation of Cx43 (Brehm et al. 2002; Brehm et al. 2006; Cheng and Mruk 2012; Tripathi and Tripathi 2010). Thus, the central role of the SC within the testis, in particular the seminiferous epithelium, is as evident as the importance of Cx43 within the SC.

Furthermore, a conditional SC-specific knockout (KO) of the *Gja1* gene (SCCx43KO, analyzed in this study) revealed Cx43 expression in SC as an absolute requirement for normal testicular development and initiation of spermatogenesis/meiosis in mice (Brehm et al. 2007; Rode et al. 2018a; Sridharan et al. 2007). Furthermore, it has been determined that a lack of Cx43 within the SCCx43KO mice leads to a partial disruption of the androgen receptor signaling pathway, which could potentially be another cause for the impaired spermatogenesis (Chojnacka et al. 2012). Interestingly, a conditional GC-specific KO of the *Gja1* gene (GCCx43KO) did not indicate the importance of Cx43 within the GC since these mice were still fertile unlike the SCCx43KO mice. Thus, proposing that the cross talk established by Cx43 between SC-GC is vital within the SC, but not the GC where it may be substituted by other Cx (Gunther et al. 2013; Rode et al. 2018b) resulting in heterotypic GJ (Koval et al. 2014; Nielsen et al. 2012).

Adult SCCx43KO (KO) mice showed normal testis descent, but testis size and weight were drastically lower when compared with heterozygous and wild-type (WT) littermates. Histological analysis revealed that SC specific deletion of Cx43 mostly resulted in an arrest of spermatogenesis at the level of undifferentiated spermatogonia or SC-only syndrome, intratubular SC cell clusters, abnormal SC cytoplasmic vacuoles, increased SC numbers, and reduced number of spermatogonia per seminiferous tubule in adult males (Brehm et al. 2007; Rode et al. 2018a; Sridharan et al. 2007; Weider et al. 2011a). Furthermore, as SC were found to be still proliferating in adult mice (Sridharan et al. 2007), it was postulated that lack of Cx43 expression in SC caused these somatic cells to remain in an apparently “intermediate” and permanent proliferative state (Weider et al. 2011a). These results emphasize the critical contribution of Cx43 to the normal maturational progression of SC, which normally results in the cessation of SC mitogenesis during the pubertal period. In contrast, Cx43 does not appear to be vital within the GC for spermatogenesis in GCCx43KO mice (Gunther et al. 2013; Rode et al. 2018b).

Several rodent SC lines have been generated during the past few decades (Wang et al. 2016). However, although investigation of SCCx43KO mice yielded important insights in the role of Cx43 in SC biology and spermatogenesis (Brehm et al. 2007; Chojnacka et al. 2012; Gerber et al. 2016; Gerber et al. 2014; Giese et al. 2012; Hollenbach et al. 2018; Rode et al. 2018a; Sridharan et al. 2007), no known KO Cx43 SC line has been established, so far. Carette et al. (2010) were successful in a partial inhibition of Cx43 in cultured SC through small interfering ribonucleic acids (siRNAs) (Carette et al. 2010), yet a complete in vitro KO of Cx43 in SC may provide beneficial results in understanding the roles of Cx43 in SC biology. Additionally, it may help to develop a mechanistic hypothesis in understanding the altered functions of Cx43 in SC leading to impaired spermatogenesis in mouse and men. Furthermore, the tight junction protein occludin showed an altered expression (at both protein and messenger ribonucleic acid (mRNA) level) in SC lacking Cx43 (Carette et al. 2010; Gerber et al. 2014; Weider et al. 2011a). It has also been noted that claudin-11, another tight junction protein, has been linked to blood-testis barrier (BTB) formation (Gow et al. 1999) and is overexpressed in men with CIS (Fink et al. 2009) and altered in arrested spermatogenesis (Chiba et al. 2012; Gerber et al. 2014; Haverfield et al. 2013; Hollenbach et al. 2018; McCabe et al. 2016; Stammler et al. 2016), concomitantly with a downregulation of Cx43 (Brehm et al. 2002; Brehm et al. 2006). Finally, in adult SCCx43KO mice, claudin-11 was found to be significantly increased at mRNA level compared with their WT littermates using real-time polymerase chain reaction (qRT-PCR) (Gerber et al. 2014), whereas claudin-3, which is supposed to play a role in BTB dynamics, was significantly downregulated (Hollenbach et al. 2018).

The objective of the present study was to develop, characterize and compare two primary SC cultures with and without Cx43. Here, with the established SCCx43KO model, testes of predominantly 17–19-day-old postpartum (p.p.) adolescent mice were used as SC of this age are supposed to be still proliferating. The isolated cells were brought into culture using a 3-step enzymatic digestion, and then the SC were subjected to different experiments: mRNA expression, immunohistochemistry (IHC), immunofluorescence (IF), semi-quantitative Western blot (WB) analysis, cell proliferation assay and cell monolayer integrity assay. The ultimate aim of the present initial characterization of the KO primary SC culture will be their transfection in order to generate an immortalized SC line lacking Cx43 to investigate its functions and involved mechanisms in SC biology while reducing the number of animal experiments.

## Materials and methods

### SCCx43KO mice

Animal experiments were approved by the animal rights committee at the regional commission of Giessen, Germany (decision V54-19c 20/15 c GI 18/1) and the regional commission of Hannover, Germany (decisions 33.9-425-05-11A120, 33.19-42502-05-16A017).

The SCCx43KO mouse line stems from crossing anti-Müllerian hormone (AMH)-Cre transgenic mice (Lecureuil et al. 2002) with floxed Cx43-LacZ transgenic mice (Theis et al. 2001; Theis et al. 2000). This is described by Brehm et al. (2007) and Sridharan et al. (2007) in greater depth. Briefly, using the SC-specific AMH promoter, the Cre recombinase enzyme is expressed under its control starting from early gestational ages until puberty (Lecureuil et al. 2002). Once expressed, Cre actively removes the Cx43-floxed genomic deoxyribonucleic acid (DNA) section. The Cx43-LacZ transgenic mice contain two loxP sites, one in front and one behind the second exon of *Gja1*, coding for Cx43. Hence, when Cre is present in the SC and both floxed alleles are deleted, a homozygous KO (SCCx43KO) mouse has been generated. Through multigenerational breeding, it is possible to achieve a 50/50 WT to KO ratio within the same litter. The genotype is determined according to the DirectPCR-Tail protocol (PEQLAB, Erlangen, Germany, 31-103-T) but with ear tissue and further analyzed via PCR as described by Brehm et al. (2007).

Adolescent mice (17–19 days old) were anesthetized with CO_2_ before being sacrificed by cervical dislocation. Testes were removed and, depending on further processing, placed into the appropriate solution/fixative as described below.

### HE staining and IHC of tissue sections

Hematoxylin and eosin (HE) staining was performed initially using Bouin-fixed tissue to determine morphology of the KO and WT mice at adolescent ages using standard techniques. Respectively, the consecutive sections were then subjected to IHC for Sox9 (also known as SRY (sex determining region Y)-box 9), Cx43, claudin-11, and vimentin, with minor changes as described previously (Brehm et al. 2007; Gerber et al. 2014). Briefly, IHC sections were pretreated with sodium citrate buffer (pH 6.0) for 20 min between 96 and 99 °C; blocked with 3% bovine serum albumin (BSA) for 20 min and incubated with the respective primary antibodies (Table 1) over night at 4 °C. The sections were then exposed for 30 min at room temperature (RT) with EnVision™ + Kit HRP Rabbit DAB+ (Dako, Hamburg, Germany, K4011), according to the manufacturer’s protocol. Tissue sections (except claudin-11) were counterstained with hematoxylin for 2 s and rinsed with running water. Finally, all sections were mounted with Eukitt® (O. Kindler GmbH, Freiburg, Germany, Eukitt). A negative control was performed by substitution of the primary antibody with buffer, and all controls were negative (data not shown). The sections were viewed under a Zeiss Axioskop microscope (Carl Zeiss, Oberkochen, Germany, ZeissAxioskop) and photographed with a DP70 Digital Camera (Olympus, Hamburg, Germany, DP70).

**Table 1:** Dilution and information of the antibodies used for immunohistochemistry (IHC), immunofluorescence (IF), and Western blot (WB)

Antibody	Host	Application	Dilution	Company	Catalog number
2nd Alexa 488	–	IF	1:5000	Invitrogen	A11008
2nd Alexa 546	–	IF	1:1000	Invitrogen	A11010
2nd Anti-Rabbit	Goat	WB	1:5000	Santa Cruz	SC-2004
Claudin-11	Rabbit	Tissue IHC	1:2000	Abcam	AB53041
		WB	1:500		
		IF	1:1000	Cohesion	CPA1843
Cx43	Rabbit	Tissue IHC	1:500	Cell Signaling	3512
		IF	1:100		
DAPI	–	IF	0.1 μg/ml	Sigma	D9542
Hoechst 33342	–	IF	1:8000	Invitrogen	H1399
Phalloidin	–	IF	1:70	Sigma	P5282
α-Tubulin	Rabbit	WB	1:1000	Cell Signaling	2125
Smooth muscle actin	Rabbit	IHC	1:200	Abcam	AB5694
Sox9	Rabbit	Cell culture IHC	1:250	Millipore	AB5535
		Tissue IHC	1:800		
		IF	1:400		
Vimentin	Rabbit	Cell culture IHC	1:250	Santa Cruz	SC-7557-R
		Tissue IHC	1:200		

### Extraction, isolation, and culture of SC

The testes were removed aseptically and placed into a test tube. Each testis was treated individually in the following 3-step enzymatic digestion, a protocol modified from Nenicu et al. (2007). This protocol for SC isolation has been modified over the past four decades (Hadley et al. 1985; Mather and Sato 1979; Monsees et al. 1996; Nenicu et al. 2007; Onoda et al. 1990; Rich et al. 1983) and was extremely successful in achieving a viable and highly pure primary SC culture. The following steps occurred under a laminar flow hood. The testis was dipped into 70% ethanol, then into 1× phosphate-buffered saline (PBS; PAA, Pasching, Austria, H15–011), and then into a new 2-ml test tube containing DMEM/Ham’sF12 medium (PAA, Pasching, Austria, E15-012). The testis was then washed with 2 ml medium two additional times, after which the tunica albuginea was removed using a surgical knife. The parenchyma was then placed into a new 2-ml test tube containing digestive medium 1 (Table 2). The sample was vortexed vigorously and then incubated and shaken for 30 min at 37 °C on a heat block shaker. The tube was then incubated for 7 min at RT to allow for gravitational sedimentation, and the supernatant was removed/discarded. Digestive medium 2 (Table 2) was added to the sediment. The sample was vortexed vigorously and then incubated at 37 °C and shaken for 15 min. Cells were sedimented by centrifuging at 50×*g* for 1 min at RT. The supernatant was removed/discarded, and digestive medium 3 (Table 2) was added. The tube was vortexed vigorously and then incubated at 37 °C and shaken for 20 min. Afterwards, sedimentation occurred via centrifugation at 100×*g* for 1 min at RT. The supernatant was then removed/discarded, and the cells were washed three times with incubation medium: 2 ml of DMEM/Ham’sF12 + 10% fetal bovine serum (FBS; PAA, Pasching, Austria, A15-151) + 1xPenicillin/Streptomycin (PAA, Pasching, Austria, P11-010) + 3 mM L-Glutamine (PAA, Pasching, Austria, M11-004). The solution was then sieved through a cell strainer with a 70 μm pore size. The cells were counted, seeded out at 50,000 cells/cm^2^ and incubated in incubation medium at 37 °C and 5% CO_2_ for 3 days. After 3 days, the cells formed a monolayer, the medium was removed, the culture was washed with 1xPBS, and subjected to a hypotonic shock to remove remaining GC: 20 mM 2-Amino-2-hydroxymethyl-propane-1,3-diol (TRIS)-HCl at a pH 7.5 for 5 min at RT (Galdieri et al. 1981). The solution was removed and the cells were incubated in incubation medium for an additional day before further experimentation occurred.

**Table 2:** Composition of the digestive medium for isolation of Sertoli cells for one testis, all enzymes stem from Sigma-Aldrich (Munich, Germany)

Digestive medium	DMEM/Ham’sF12	Collagenase (C0130)	DNase (DN25)	Hyaluronidase (H3506)
1	1.25 ml	1 mg/ml	20 μg/ml	–
2	1.25 ml	2 mg/ml	20 μg/ml	2 mg/ml
3	1.25 ml	2 mg/ml	20 μg/ml	2 mg/ml

### Characterization of SCCx43KO primary cell cultures via morphology, IHC, and IF

Initial characterization of the primary SCCx43KO cells was visually assessed through the morphological character of the SC via phase contrast microscopy at day 4 of culture.

To begin with, vimentin, an intermediate filament, was used for SC characterization (IHC). However, since vimentin is expressed in all mesenchymally derived cells (thus, also e.g., in Leydig cells and peritubular cells), an additional marker was required. Hence, the SC specific nuclear transcription factor Sox9 was used to further characterize the primary SCCx43KO cell cultures (IHC, IF). The purity of the SC within the primary cell cultures on day 4 of culture (1 day after hypotonic shock) was determined using Sox9 IHC; positive nuclei were identified as SC nuclei. More than 1000 cells per mouse were analyzed and from each genotype, three cultures were assessed.

Conversely, smooth muscle actin (SMA) was used to depict remaining peritubular cells in the SC culture to visualize SC purity.

Finally, the presence or absence of Cx43 and claudin-11 protein in both KO and WT SC cultures was assessed by IF.

The primary cell cultures were seeded out onto glass cover slides with a concentration of 50,000 cells/cm^2^ in 24-well plates. On day 4 of culture, the medium was removed and the cultures were washed twice with either Tris-buffered saline (TBS) solution (vimentin, Sox9) or PBS (Cx43, claudin-11). The cells were then fixated with either 1 ml methanol for 10 min (claudin-11, vimentin, Sox9, SMA) or 3% paraformaldehyde for 4 min (Cx43) at RT. Afterwards, the fixative was removed and the cells were washed with TBS-Tween (TBST) and then blocked on a shaker using either 5% non-fat dry milk in TBST for 45 min (vimentin, Sox9, SMA) or 3% BSA diluted in PBS for 30 min (Cx43, claudin-11) at RT. The solution was removed and the primary antibody (Table 1), diluted in TBS (Sox9, vimentin, SMA) or PBS containing 1% BSA (Cx43, claudin-11), was added and incubated overnight at 4 °C.

For IHC (vimentin, Sox9, SMA), on the next day the cells were washed three times with TBS for 10 min each, and visualization occurred according to a slightly modified EnVision+ System-HRP-DAB-Rabbit Kit protocol (Dako, Hamburg, Germany, K4011). Briefly, the labeled Polymer-HRP Anti-Rabbit was added for 20 min, and then washed three times with TBS for 5 min. DAB+ Substrate Buffer and DAB+ Chromogen solution (mixed according to protocol) was added for 5 to 10 min. The cells were then washed for 5 min with water. Note, for better visualization only the vimentin and SMA IHC assays were counter-stained with hematoxylin to identify the nuclei. The glass cover slides were then fixated with gelatin onto a microscope slide. Stains were performed in triplicate for each genotype; negative controls were performed by omitting the primary antibody from the TBS solution. The cells were viewed under a Zeiss Axioskop microscope (Carl Zeiss, Oberkochen, Germany, ZeissAxioskop) and photographed with a DP70 Digital Camera (Olympus, Hamburg, Germany, DP70).

For Sox9, Cx43, and claudin-11 IF, the second day, the cells were washed three times either in TBS for 10 min each (Sox9) or in PBS for 5 min each (Cx43, claudin-11), and the secondary antibody, Alexa 488 (Sox9) or Alexa 546 (Cx43, claudin-11) respectively (Table **1**), was added for 45 min at RT diluted in either TBS (Sox9) or PBS containing 1% BSA (Cx43, claudin-11). The cells were washed three times for 5 min with TBS (Sox9) or PBS (Cx43, claudin-11). To stain the nuclei, either Hoechst 33342 (Table **1**) diluted in TBS was added for 5 min at RT (Sox9) or slides were incubated with DAPI (Table **1**) diluted in PBS for 10 min at RT (Cx43, claudin-11). For the Cx43 IF, F-actin, a cytoskeletal protein, was visualized using phalloidin (Table **1**) in order to better identify the cell borders. The cells were then washed for 5 min with water (Sox9) or three times for 5 min each in PBS (Cx43, claudin-11). Glass cover slides were then fixated with ProLong® Gold Antifade Reagent Antifade (Thermo Fisher Scientific, Darmstadt, Germany, P36930) onto a microscope slide. The cells were viewed under a Zeiss Axiovert 200M fluorescence microscope (Carl Zeiss, Oberkochen, Germany, Zeiss Axiovert 200M). A negative control was performed by omitting the primary antibody from the TBS/PBS solution.

Quantification of claudin-11 fluorescence was performed using ImageJ (version 1.51.0) by determining the mean gray value of pictures and subtracting the mean gray value of the background. Subsequently, the number of primary SC in the picture was determined and the mean gray value (mean fluorescent intensity) per SC was calculated. At least 200 cells per genotype of three cell culture passages were analyzed. Significance was determined using a Student’s *t* test and a *p* value of < 0.05 was defined as significant with *p < 0.05.

### Characterization of mRNA expression

The cells were harvested and the RNA was isolated according to the PureLink™ RNA Mini Kit (Thermo Fisher Scientific, Darmstadt, Germany, 12183018A) protocol. Briefly, after washing the cells with PBS, they were detached using trypsin for 5 min at 37 °C and transferred to a 15-ml test tube. The cells were pelleted through centrifugation and were incubated with lysis buffer and vortexed. The suspension was then homogenized using the T 10 basic ULTRA-TURRAX® (IKA, Staufen, Germany, 3737000) with the S10N-5G adaptor (IKA, Staufen, Germany, 3304000) at maximum speed for 45 s. The samples were then centrifuged at 21,000×*g* for 5 min, and the supernatant was transferred to a new tube and further processed. The samples were vortexed with 70% ethanol and were then transferred to a spin cartridge. Next, they were subjected to centrifugation at 12,000×*g* for 15 s and the flow through was discarded. The spin cartridge was then washed with wash buffer I and centrifuged under the same conditions. At this point, a DNA digest was performed using the PureLink™ DNase (Thermo Fisher Scientific, Darmstadt, Germany, 12185010) and incubated for 15 min. The samples were then centrifuged at 12,000×*g* for 15 s, and the flow through was discarded. They were then washed twice with wash buffer II, centrifuged at 12,000×*g* for 15 s, and the flow through was discarded after each step. The mRNA was released from the spin cartridge through the addition of RNAse-free water and centrifuged at 12,000×*g* for 2 min.

The mRNA samples were then transcribed into complementary DNA (cDNA) using a reverse transcription PCR (RT-PCR) according to the TaqMan® Gold RT-PCR Kit, Reverse Transcription, using the MultiScribe Reverse™ Transcriptase (Thermo Fisher Scientific, Darmstadt, Germany, 4311235). PCR of the cDNA was performed to characterize the primary cell culture according to the GoTaq® Flexi DNA Polymerase (Promega, Mannheim, Germany, M8307) protocol using the specific primers (Table 3).

**Table 3:** List of genes, their respective primer sequences, and amplification lengths for PCR reactions. All primers were purchased from Eurofins MWG (Eurofins MWG, Ebersberg, Germany)

Gene	Direction	Primer sequences	Length (bp)	Source
*Acta2*	Forward Reverse	AATGAGATGGCCACGGCCGCGTCTCTGGGCAGCGGAAGCG	107	Self-design
*Actb*	Forward Reverse	TTCCTTCTTGGGCATGGAGTTACAGGTCTTTGCGGATGTC	90	Weider et al. 2011a
*Amh*	Forward Reverse	CCAACGACTCCCGCAGCTCCTTCCCGCCCATGCCACTC	93	Weider et al. 2011a
*Cldn11*	Forward Reverse	CGTCATGGCCACTGGTCTCTGGCTCTACAAGCCTGCACGTA	82	Giese et al. 2012
*Gja1*	Forward Reverse	ACAGCGGTTGAGTCAGCTTGGAGAGATGGGGAAGGACTTGT	106	Giese et al. 2012
*Hsd3b6*	Forward Reverse	GTGCTGGCTTTGCTTCCCCCTCGCTCCACCCAGGCATGGTCAAC	333	Self-design
*Hsp90ab1*	Forward Reverse	AAGAGAGCAAGGCAAAGTTTGAGTGGTCACAATGCAGCAAGGT	120	Weider et al. 2011b
*Ocln*	Forward Reverse	ATCCTGTCTATGCTCATTATTGTGCTGCTCTTGGGTCTGTATATCC	205	Giese et al. 2012
*Sox9*	Forward Reverse	CGGAGGAAGTCGGTGAAGAGTCGGTTTTGGGAGTGGTG	201	Barrionuevo et al. 2006
*Tjp1*	Forward Reverse	CCCTACCAACCTCGGCCTTAACGCTGGAAATAACCTCGTTC	97	Giese et al. 2012

### Semi-quantitative WB analysis

Protein extraction for WB analysis was performed according to the CelLytic™ MEM Protein Extraction Kit (Sigma-Aldrich, Munich, Germany, CE0050) protocol. Briefly, testes were homogenized as described in the RNA isolation section using the lysis solution. The cultured cells were detached using a cell scraper, pelleted via centrifugation at 600×*g* for 5 min, and the supernatant was removed. The samples (from the cell culture and homogenized testes) were incubated in lysis solution for 10 min on ice. Afterwards, the samples were centrifuged at 4 °C at > 10,000×*g* for 5 min. The supernatant, containing the protein, was removed. The proteins were separated via sodium dodecyl sulfate-polyacrylamide gel electrophoresis (SDS-PAGE; gel concentration 12%) and then blotted onto a nitrocellulose membrane.

The membrane was then blocked in 5% non-fat dry milk in TBST for 45 min on a shaker (membrane always shaken during incubation from this step on). The membrane was then incubated overnight at 4 °C with the primary antibody for claudin-11 (Table 1) diluted in TBS. The next day, the membrane was washed four times in TBST for 5 min each, and the respective secondary antibody (Table 1) was incubated at RT for 45 min. Afterwards, the membrane was washed three times for 5 min with TBST and once for an additional 5 min with TBS. The chemiluminescence was visualized via the SuperSignal® West Dura Extended Duration Substrate (Thermo Fisher Scientific, Bonn, Germany, 34076).

To detect the loading control on the membrane, the blot was washed twice in TBS for 5 min, twice in stripping buffer for 30 min, and then washed four times in TBST for 5 min. The steps from the previous paragraph were repeated for the respective housekeeper (Table 1). Note all antibodies were tested for false positives by omitting the primary antibody. Significance was determined using a Student’s *t* test and a *p* value of < 0.05 was defined as significant with *p < 0.05.

### Cell proliferation assay

The proliferation assay was performed to determine if there was any proliferation in the KO and WT primary SC cultures. This colorimetric assay is based on a conversion of the pale yellow tetrazolium salt 3-(4,5-dimethylthiazol-2-yl)-2,5-diphenyltetrazolium to the dark blue formazan by any living cells. From the optical density (OD) of the formazan solution, it can be extrapolated to the number of living cells meaning the higher the OD of the formazan solution, the more viable cells are in the cell culture (Mosmann 1983). The isolated cells from each mouse were seeded out in triplicate onto 96-well plates at a concentration of 50,000 cells/cm^2^. The cultures were analyzed on days 4, 6, and 8 (1, 3, and 5 days after hypotonic shock, respectively); after seeding out, the incubation medium was removed and a 1:9 mixture of MTT-Solution and DMEM/Ham’sF12 medium was added (100 μl for each 96-well) and incubated for 1 h at 37 °C and 5% CO_2_. The MTT solution consists of 5 mg of 3-(4,5-dimethylthiazol-2-yl)-2,5-diphenyltetrazolium bromide (Thermo Fisher Scientific, Darmstadt, Germany, M-6494) diluted in 1 ml of 1× PBS. After incubation, the solution was removed and the same volume of dimethyl sulfoxide was added and shaken for 15 min at RT. Measurements were taken using a Multiskan EX and the respective Ascent Software (Thermo Scientific, Bonn, Germany, 51118170). The differences between 550 nm and 690 nm measurements were then calculated.

### Measurement of cell monolayer integrity

Cultured SCs are capable of forming an intact epithelium with a functional TJ-permeability barrier (Mruk and Cheng 2011). By measuring the transepithelial electrical resistance (TEER) across such a cell monolayer using a bicameral system, its cell monolayer integrity is determined (Mruk and Cheng 2011; Srinivasan et al. 2015). Using TEER measurements in KO and WT primary SC cultures, differences in tight junction expression were determined. The cells were seeded out at 50,000 cell/cm^2^ onto a 0.4 μm ThinCert™ (Greiner Bio-One, Frickenhausen, Germany, 665,641) and incubated in a well of a 12-well plate. The insert contained 0.5 ml and the well 1.5 ml of incubation medium. TEER measurements were taken daily from day 0 to day 8 of culture using an EVOM voltmeter (World Precision Instruments, Berlin, Germany, EVOM) with a STX2 electrode (World Precision Instruments, Berlin, Germany, STX2). Measurements were performed in triplicate each day. After day 3 of culture, medium was changed every 2 days. On the days when medium change occurred, measurements were taken after a minimum incubation time of 30 min to eliminate possible temperature dependent fluctuations. All measurements were normalized to a blank well containing a ThinCert™ and incubation medium. Statistical analysis was performed via a one-way ANOVA test using the software SPSS version 15.0 (IBM SPSS Statistics, Ehningen, Germany). A *p* value of < 0.05 was defined as significant with *p < 0.05.

## Results

### Histology and IHC of testicular tissue of adolescent and adult mice

**Fig. 1:**
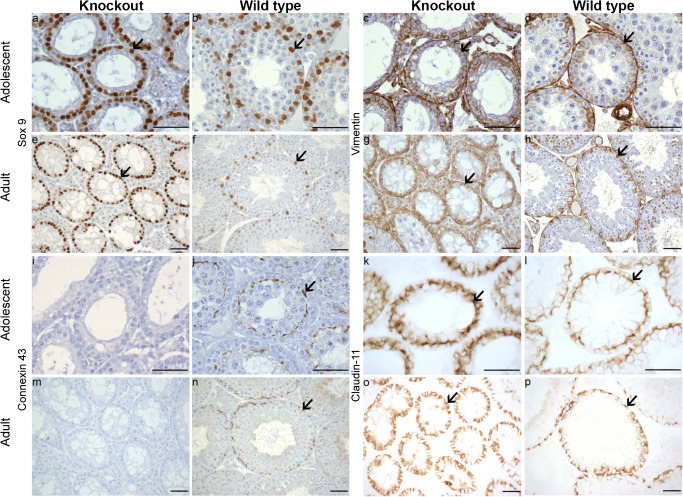
Representative immunohistochemical stainings (Sox9, vimentin, connexin43 (Cx43), and claudin-11) from adolescent (17–19 days postpartum) and adult knockout (KO) and wild-type (WT) testes. A total of *n* = 8 KO and *n* = 7 WT adolescent mice were investigated, and at least one KO and WT adult mouse was used as a control. Sox9 is a Sertoli cell (SC) specific nuclear marker indicated by the arrows in a, b, e, and f. Vimentin is a SC, peritubular and Leydig cell cytoplasmic marker and is indicated by the arrows in c, d, g, and h. Note that the adult testes express an unspecific binding of the vimentin antibody in the round spermatids in some tubules. Cx43 is a gap junction protein located at the height of the blood-testis barrier between adjacent SC and between SC and germ cells and is indicated by the arrows in j and n. The KO testes express no Cx43 (i and m). Claudin-11 (without counterstaining) is a tight junction protein that is located between two SC and is indicated by the arrows in k, l, o, and p. Scale bars 50 μm

Preliminary testing was performed using testicular tissue via HE staining and IHC for Sox9, vimentin, Cx43, and claudin-11 from both adolescent and adult KO and WT mice and revealed normal spermatogenesis in WT mice, while the KO mice showed impaired spermatogenesis (Fig. 1 and Supplementary Videos S1, S2, S3, and S4). Specifically, the adolescent WT mice show the beginning “wave(s)” of spermatogenesis up to spermatocytes (Fig. 1b, d, j, l). Nuclear Sox9 immunostaining in the adolescent and adult KO mice depict SC rich tubules with few to no GC (Fig. 1a, e), while the adolescent and adult WT mice exhibit several SC with more GC per tubule than in the KO model (Fig. 1b, f). The intermediate filament vimentin, localized in the cytoplasm of mesenchymally derived cells, is evident in SC, Leydig cells and peritubular cells of both adolescent and adult KO and WT mice (Fig. 1c, d, g, h). Note for some unknown reasons, the vimentin antibody also stained the nucleus of some round spermatids in the adult WT mice (Fig. 1h); this immunoreaction should be considered as nonspecific. Cx43 immunostaining is located at the height of the BTB in the WT of both adolescent and adult mice (Fig. 1j, n) and was absent in KO males (Fig. 1I, m). Claudin-11 (without counterstaining) forms a diffuse band during the adolescent ages and localizes towards the BTB at the adult age in both genotypes (Fig. 1k, l, o, p). Finally, through HE staining, it was evident that seminiferous tubules of adolescent mice contain SC mitotic figures visualized through vimentin and Sox9 immunolabeling (Supplementary Videos S1, S2, S3, and S4).

In preliminary experiments, adult mice were chosen for initial cultures, yet approximately half of the isolations could be successfully sustained in culture after the 3-step enzymatic digestion and hypotonic shock (data not shown). Due to these viability issues, adolescent 17–19-day-old mice were then chosen, since many publications used a similar age bracket for successful SC cultures in rats and mice (Hadley et al. 1985; Monsees et al. 1996; Nenicu et al. 2007; Onoda et al. 1990). Additionally, SC mitotic figures as seen in the Supplementary Videos 1–4 during the adolescent ages were of interest for a possible proliferative SC line.

### Analysis of the 3-step enzymatic digestion

**Fig. 2:**
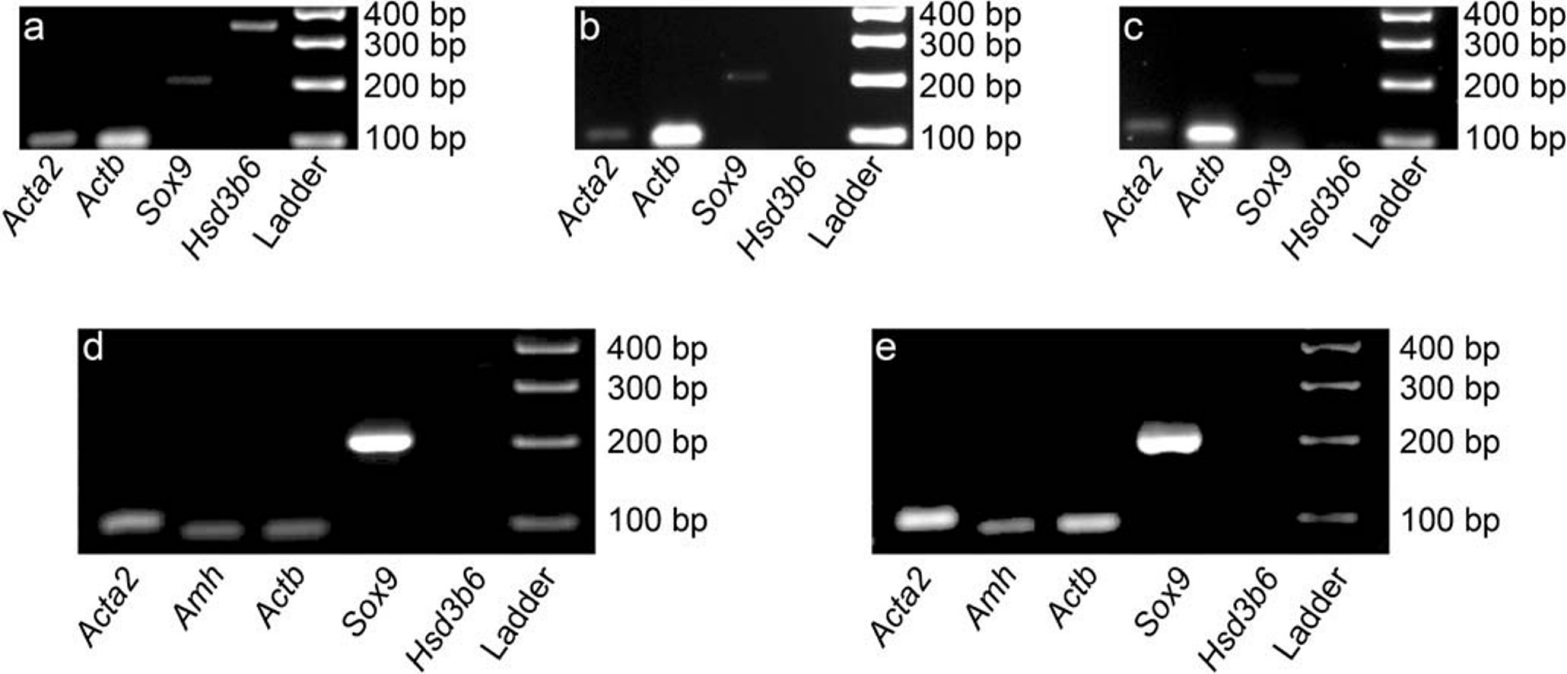
Representative gel electrophoresis/PCR analysis of transcribed cDNA (*n* = 3 knockout (KO); *n* = 3 wild type (WT)), from supernatant removed/discarded after each digestive step 1–3 (**a**–**c**, respectively, no difference between KO and WT genotypes could be determined; images stem from a KO mouse) and the primary cell cultures 4 days after seeding out (**d** = KO and **e** = WT). *Actb,* which codes for β-actin, was used as a positive control for **a**–**e**. **a** First discarded supernatant contained peritubular myoid cells (*Acta2*), SC (*Sox9*), and Leydig cells (*Hsd3b6*). **b** Second discarded supernatant contained peritubular myoid cells (*Acta2*) and SC (*Sox9*). **c** Third discarded supernatant contained peritubular myoid cells (*Acta2*) and SC (*Sox9*). **d** KO and **e** WT: primary cell cultures contained traces of peritubular myoid cells (*Acta2*), SC maturation marker (*Amh*) and SC (*Sox9*), yet no traces of Leydig cells (*Hsd3b6*)

To determine the effectiveness of the 3-step enzymatic digestion, the RNA of the supernatant after sedimentation was purified. The cDNA from cells in the supernatant was then characterized using specific primer sequences (Table 3). Figure 2a depicts the removal of peritubular myoid cells (*Acta2* (Cool et al. 2008, Hofmann et al. 2003)), SC (*Sox9* (Frojdman et al. 2000; Graves 1998; Hemendinger et al. 2002; Kent et al. 1996)), and Leydig cells (*Hsd3b6* (Baker et al. 1999, O'Shaughnessy et al. 2002)) after the first digestive step. Figure 2a and c correspond to the removal of peritubular cells and SC, while no Leydig cells were removed after the second and third digestive step, respectively. *Actb*, which codes for β-actin, was used as a positive control. In conclusion, the first digestive step removed the Leydig cells, while the second and third exclusively purified a few peritubular myoid cells from the SC primary culture.

### RNA characterization of the primary cell culture

To ensure that the cultured cells were SC, RNA from the primary cell culture was characterized 1 day after hypotonic shock (day 4 of culture). This shock is a selective removal of the GC, which has no effect on the SC viability (Galdieri et al. 1981). It is evident that both KO and WT cultures contain RNA from peritubular cells (*Acta2*) and SC (*Sox9*, *Amh*), yet no Leydig cells (*Hsd3b6*) (Fig. 2d, e). *Actb,* which codes for β-actin, was used as a positive control, and the SC prepubertal marker *Amh* was used in addition to the SC marker *Sox9*. As the cultured SC were harvested from adolescent mice, *Amh* showed only a weak band since the expression of *Amh* by SC ceases during puberty (Al-Attar et al. 1997; Munsterberg and Lovell-Badge 1991). The RNA characterization of the primary cell culture depicts a mixture of peritubular cells (*Acta2*) and SC (*Amh*, *Sox9*).

### Visualization of the primary cell culture

**Fig. 3:**
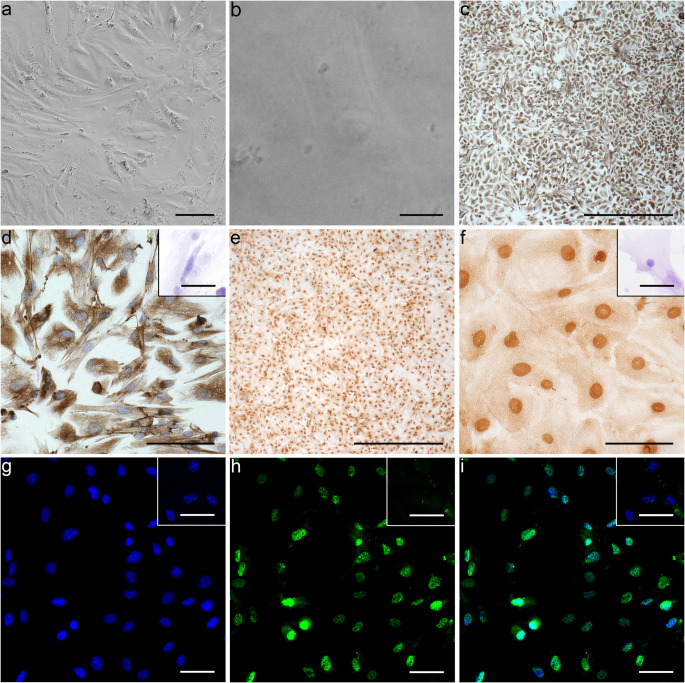
Representative microscopic pictures of the primary Seroli cell (SC) cultures. **a**, **b** Phase contrast microscopy (scale bar 100 μm and 10 μm, respectively). **a** Stems from a KO mouse, while **b**–**i** stem from WT mice. No visual differences could be determined between KO and WT during any of the staining processes. **c**, **d** (*n* = 3 KO; *n* = 3 WT): immunohistochemical staining of vimentin (brown), cytoplasmic intermediate filament detection of mesenchymally derived cells and counterstained with hematoxylin (scale bar 1000 μm and 100 μm, respectively). Inset in **d** shows the negative control of vimentin and counterstained with hematoxylin (scale bar 50 μm). **e**, **f** (*n* = 3 KO; *n* = 3 WT): immunohistochemical staining of the SC specific transcription factor Sox9 (brown; scale bar 1000 μm and 100 μm, respectively). Inset in **f** shows the negative control of Sox9 and counterstained with hematoxylin (scale bar 50 μm). **g**–**i** (*n* = 1 KO; *n* = 1 WT): IF staining of Sox9 (scale bar 50 μm). **g** Hoechst 33342 staining (blue) and nucleic acid detection. **h** Sox9 staining (green). **i** Merged image of **g** and **h**. Insets in **g**–**i** show the negative control of Sox9 (scale bar 50 μm)

The primary cell cultures were photographed 1 day after (day 4 of culture) the hypotonic shock via phase contrast microscopy (Fig. 3a, b). Figure 3a and b show the characteristic structures of SC in culture with their typical elongated cytoplasmic extensions and the distinctive nucleus located in the center of the cells (spindle shape). This spindle shape is particularly evident in Fig. 3b. It was evident after the 3-step enzymatic digestion and hypotonic shock that both KO and WT primary SC cell cultures formed monolayers when seeded out at ~ 50,000 cells/cm^2^. As performed by Nenicu et al. (2007), the intermediate filament vimentin was used to characterize mesenchymal cells (peritubular myoid cells, SC and Leydig cells) in the culture (Fig. 3i, c, d). All cultured cells were stained positive for vimentin, which is located throughout the cytoplasm. Since the purity of SC in the primary cell culture could not be determined only through vimentin, SC specific Sox9 (Frojdman et al. 2000, Graves 1998, Hemendinger et al. 2002, Kent et al. 1996) was used (Fig. 3e, f). This factor is critical for SC and male differentiation and actually precedes *Amh* expression, which was used as a prepubertal SC marker in Fig. 2d, e (Morais da Silva et al. 1996; Oreal et al. 1998). Again, the vast majority of cells appeared immunopositive for Sox9, yet a double staining was required to ensure that all cells were SC. Using IF, it was additionally possible to determine the purity of the primary SC culture (Fig. 3g–i). The cells’ nucleic acid was stained blue via Hoechst and the nuclear Sox9 of SC was stained green (Fig. 3g, h, respectively). The overlay of both images indicated a high purity of SC in the primary cell culture (Fig. 3i). Depiction of SMA in peritubular cells confirmed the purity of the SC cultures as only few cells were immunopositive for SMA (Supplemental Fig. S1). No differences between the WT and KO genotypes could be determined in any of the staining processes. All of the insets in Fig. 3 depict the successful negative controls of each primary antibody respectively.

**Fig. 4:**
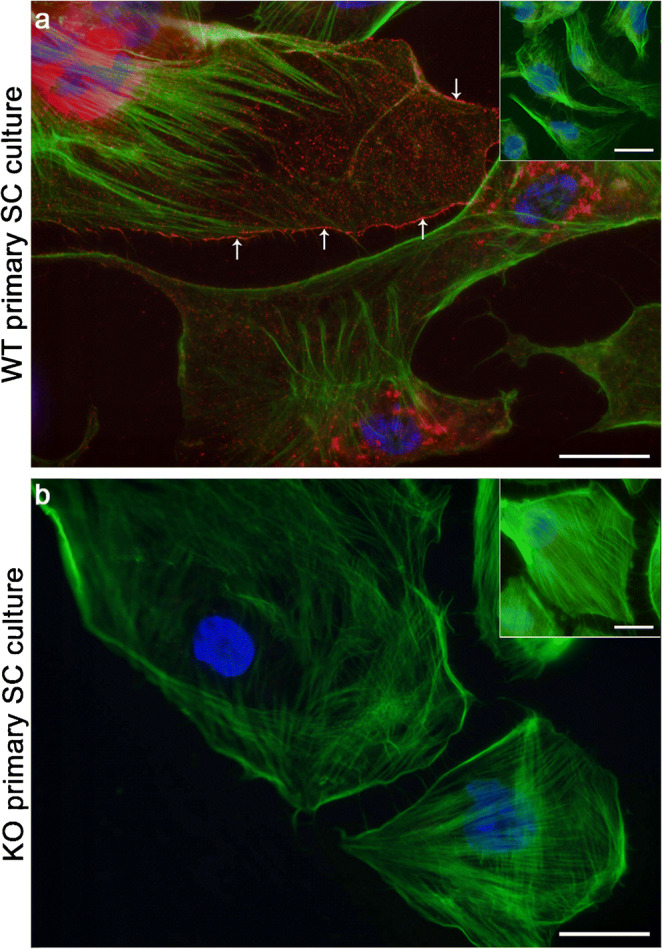
Representative images of connexin43 (Cx43) immunofluorescence of knockout (KO) and wild-type (WT) primary Sertoli cell (SC) cultures at day 4 of culture. In WT SC (**a**), Cx43 (red staining) is detectable in the cytoplasm (especially around the nucleus) and at the cell surface (arrows), while it is absent in KO SC cultures (**b**). Nuclei are stained blue (DAPI) and F-actin is stained green (phalloidin). Insets in **a** and **b** show representative negative controls. Scale bars 20 μm

In order to confirm that Cx43 protein is present in WT and absent in KO primary SC cultures, IF of Cx43 was performed at day 4 of culture. As shown in Fig. 4a, the WT SC culture was immunopositive (Fig. 4, red staining), with Cx43 being located both in the cytoplasm especially around the SC nuclei and as small dots at the cell surfaces (Fig. 4, arrows). The immunolocalization of Cx43 is in accordance with previous observations (Lablack et al. 1998), who also reported that the cytoplasmic appearance of Cx43 seems to be associated with the Golgi apparatus possibly reflecting different steps of gap junction formation. This was not the case for cultured KO SC as Cx43 was absent in these cell cultures (Fig. 4b).

### Purity of the primary cell culture

The purity of the SC within the primary cell cultures on day 4 of culture (1 day after hypotonic shock) was quantified based on the Sox9 IHC of Fig. 3. More than 1000 cells per mouse were counted and from each genotype; three cultures were analyzed. It was determined that the primary cell cultures contained a SC purity > 98%. Sox9 was expressed in RNA (Fig. 2d, e), detected in IHC (Fig. 3e, f) and IF (Fig. 3g–i). In order to double-check SC purity conversely, very few peritubular cells were identified by immunolocalization of SMA (Supplemental Fig. 1) confirming a highly pure primary SC culture.

### Cell proliferation assay

**Fig. 5:**
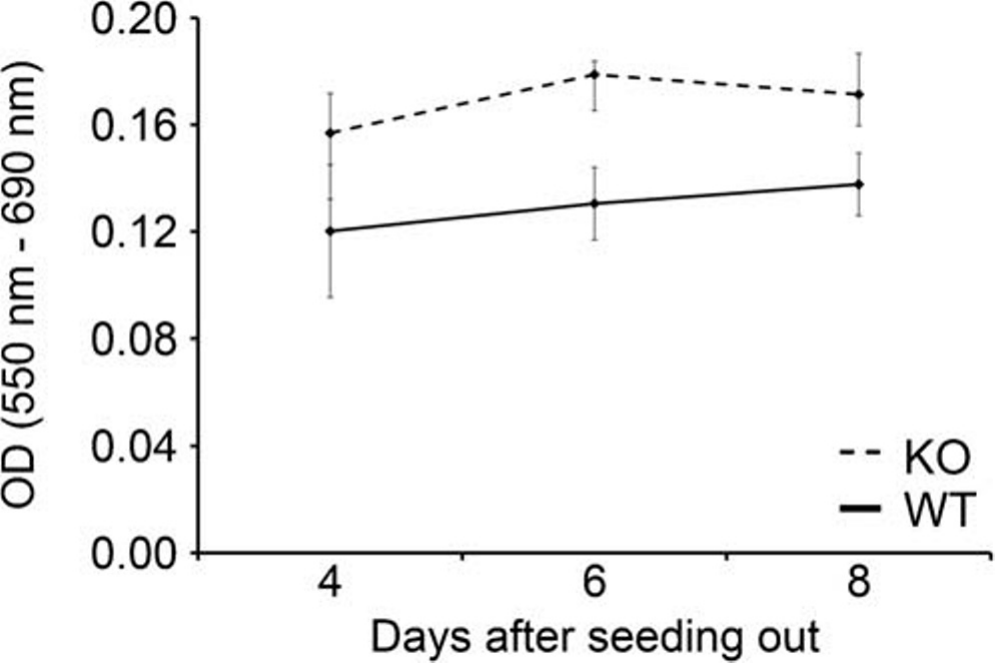
Graph of cell viability assay MTT measurements, for each mouse three separate cultures were seeded out at 50,000 cell/cm2 and were measured for their optical density at 690 nm and 550 nm (day 4: *n* = 9 wild type (WT); *n* = 7 knockout (KO); day 6 and day 8: *n* = 3 WT; *n* = 3 KO)

The proliferation of the primary cell cultures were measured on days 4, 6, and 8 of culture (1, 3, and 5 days after the hypotonic shock, respectively) and compared between KO and WT cultures. The assay is based on a conversion of 3-(4,5-dimethylthiazol-2-yl)-2,5-diphenyltetrazolium to by any living cells (Mosmann 1983). From the OD of the formazan solution, it can be extrapolated to the number of living cells, meaning the higher the OD of the formazan solution, the more viable cells are in the cell culture. In both the KO and WT primary SC cultures, there is no significant difference of the OD between day 4 and day 8 of culture (Fig. 5) indicating that the cells are viable, but do not proliferate significantly between days 4 and 8 of culture.

### Cell monolayer integrity

**Fig. 6:**
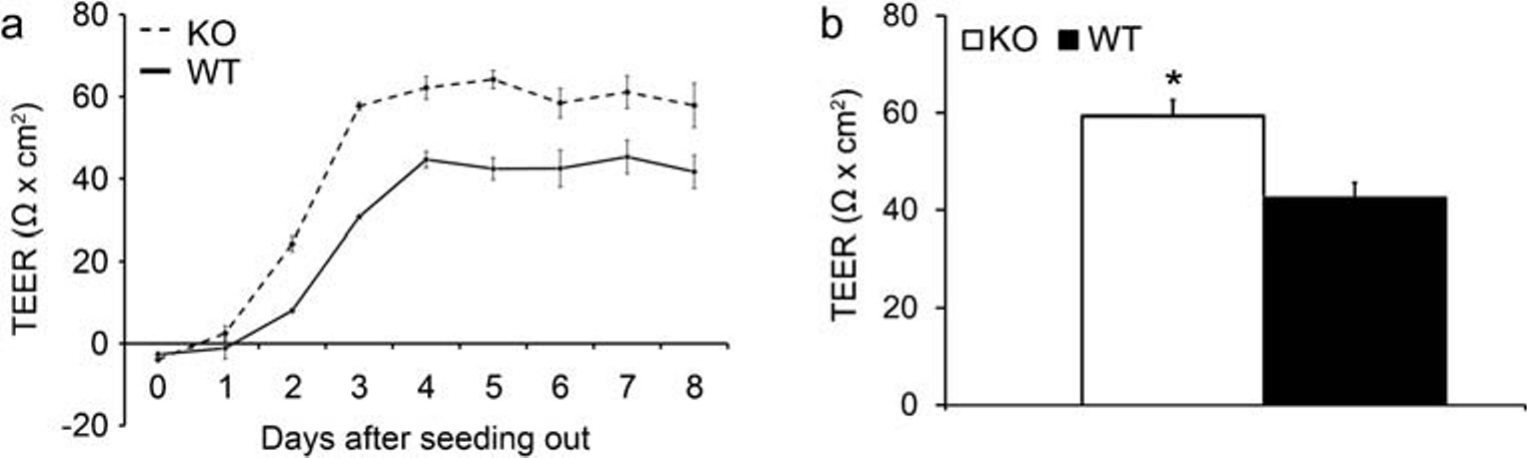
Graph of the cell monolayer integrity: transepithelial electrical resistance (TEER) measurements from primary cell cultures seeded out at 50,000 cell/cm^2^. Measurements were taken in triplicate daily over a minimum of 8 days. Individual averaged daily measurements are seen in **a**, while an average over days 4–8 is seen in **b**. All measurements were subtracted from a blank well containing the same insert, medium and volume of medium and multiplied by the insert cell growth area (*p < 0.05) (days 1–3: *n* = 3 wild type (WT); *n* = 3 knockout (KO); days 4–8: *n* = 7 WT; *n* = 6 KO)

To determine the cell monolayer integrity, the primary cell cultures were measured for TEER once a day in triplicate from day 0 to day 8 of culture (Fig. 6a). The average over the 5 d (between days 4 and 8 of culture) measurements can be seen in Fig. 6b. The measurements on day 3 were taken before the hypotonic shock was administered. The TEER values increased during the first 3–4 days of culture until followed a plateau for the next 4–5 days (Fig. 6a). The higher the TEER, the more tight junctions are present in a cell monolayer indicating that TJ establishment took place during the first 3 (KO primary SC) or 4 (WT primary SC) days of culture and the TJ were maintained for the following days of culture (Fig. 6a). These measurements not only indicated a significant increase in tight junction formation in KO cultures in comparison to the WT cultures (Fig. 6b) but also showed an earlier TJ formation in the KO cultures compared to WT cultures (Fig. 6a).

### mRNA tight junction expression

**Fig. 7:**
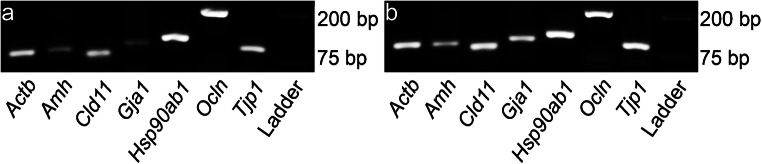
Representative gel electrophoresis PCR analysis of transcribed cDNA (*n* = 3 knockout (KO); *n* = 3 wild type (WT)), from primary cell cultures 4 days after seeding out (**a** = KO and **b** = WT). *Actb* and *Hsp90ab1,* which code for beta-actin and heat shock protein 90  kDa alpha (respectively), were used as positive controls for **a** and **b**. The following sequences were detected: Sertoli cell maturation marker (*Amh*), claudin-11 (*Cldn11*), Cx43 (*Gja1*), occludin (*Ocln*), and ZO-1 (*Tjp1*)

After determining a significantly higher electrical resistance in the KO primary cell cultures, identification of mRNA of the junction components *Cldn11* (claudin-11), *Gja1* (Cx43), *Ocln* (occludin), and *Tjp1* (zonula occludens-1, ZO-1) was performed on day 4 of culture 24 h after hypotonic shock (Fig. 7) using RT-PCR. *Amh* (AMH) was used as a SC marker. The housekeepers *Actb* (β-actin) and *Hsp90ab1* (heat shock protein 90 kDa alpha) were used as controls. mRNA of all investigated tight junction proteins could be detected in both KO and WT SC cultures. At RNA level, a weak band for *Gja1* could be detected via RT-PCR (Fig. 7a) probably originating from peritubular cells as already described (Risley et al. 1992).

### Semi-quantitative WB analysis of claudin-11

**Fig. 8:**
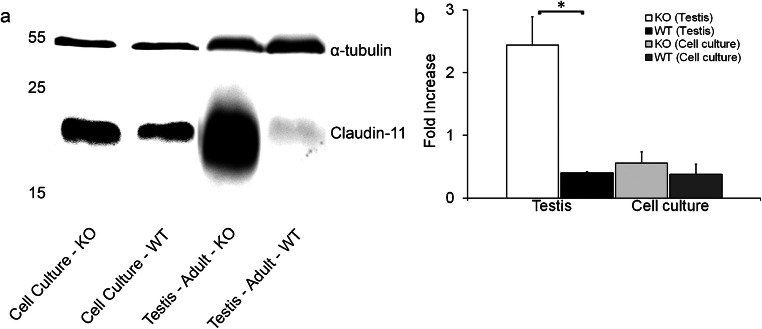
Representative Western blot (WB) of claudin-11 (*n* = 3 knockout (KO); *n* = 3 wild type (WT)). Proteins were isolated from primary cell cultures 4 days after seeding out and adult testes homogenate. α-Tubulin (52 kDa) was used as loading control and housekeeper for all blots. **a** Claudin-11 (22 kDa) was synthesized in the primary Sertoli cell (SC) cultures and adult testes homogenate of both genotypes. **b** Respective semi-quantitative analysis for claudin-11 synthesis in primary SC cultures and adult testes homogenate showed a significantly increased amount of claudin-11 protein in adult testes homogenate of KO mice compared to age-matched WT mice. However, no significant difference between genotypes could be determined comparing claudin-11 protein levels of the primary SC cultures (*n* = 3 KO; *n* = 3 WT; *p < 0.05)

To further investigate TEER results at the protein level, a WB of the tight junction protein claudin-11 was performed on day 4 of culture 24 h after hypotonic shock (Fig. 8a). The loading control α-tubulin was used to ensure that equal amounts of protein were loaded into each well. A semi-quantitative analysis determined no significant increase of claudin-11 between KO and WT cell culture samples (*p* > 0.05; Fig. 8b), yet a trend appeared that the KO mice synthesized more tight junctions than their WT littermates in the primary cell cultures. Nevertheless, the testes homogenates of adult mice revealed a significant increase in claudin-11 synthesis in the KO mice testis (*p* < 0.05); Fig. 8a and b).

### IF of claudin-11 in primary SC cultures

**Fig. 9:**
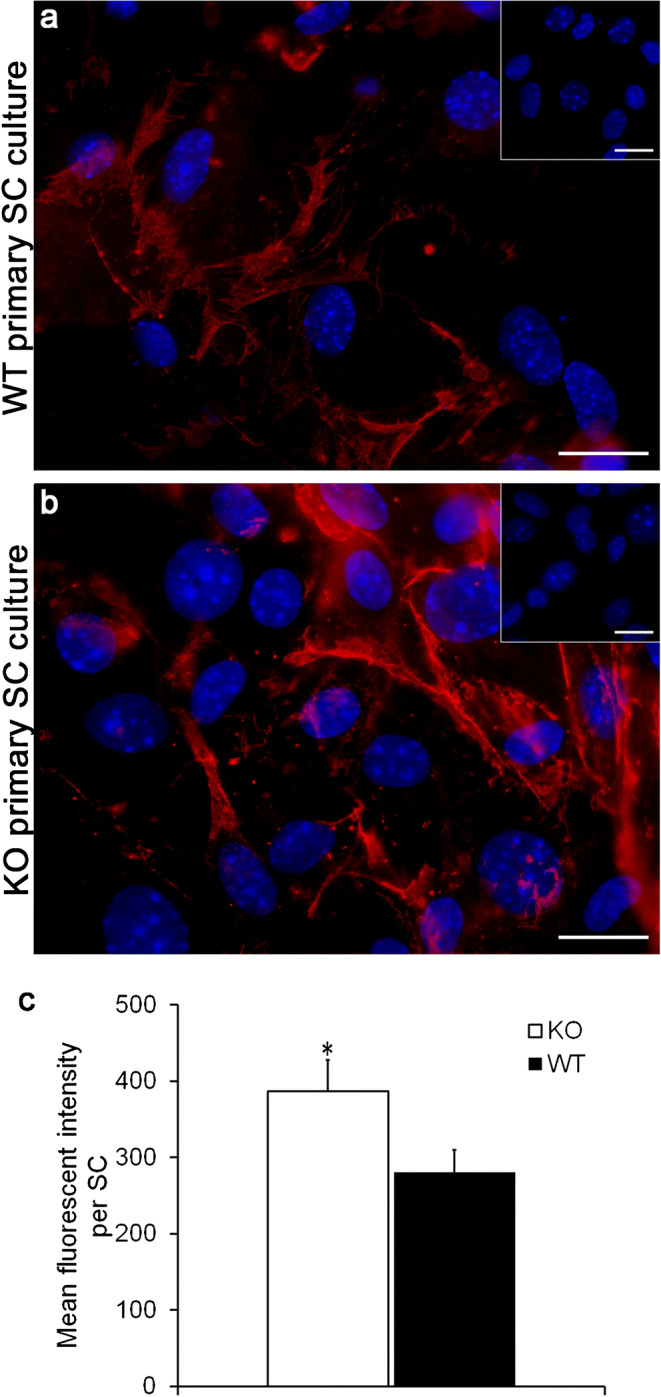
Representative images of claudin-11 immunofluorescence of knockout (KO) and wild-type (WT) primary Sertoli cell (SC) cultures at day 4 of culture. In both WT (**a**) and KO (**b**) SC, claudin-11 (red staining) is detectable along cell surface at the contact sites of adjacent SC. Mean fluorescent intensity per SC was significantly increased in KO primary SC compared to WT SC (**c**). Nuclei are depicted in blue color (DAPI). Insets in **a** and **b** show representative negative controls (*p < 0.05; scale bars 20 μm)

Using IF, claudin-11 protein synthesis and localization in primary SC cultures (KO vs. WT) at day 4 of culture was visualized to complement further generated data of TEER analysis, RT-PCR, and semi-quantitative WB regarding barrier function and tight junction synthesis. Claudin-11 was mainly localized along the SC membranes, forming contact areas between adjacent SC, in both KO and WT SC cultures (Fig. 9a and b). Furthermore, Cx43 KO SC seemed to produce more claudin-11 protein compared to WT primary SC (Fig. 9a and b) resulting in a significantly increased mean fluorescent intensity per SC (*p* < 0.05) in KO cell cultures compared to WT cultures (Fig. 9c), which is in accordance with previous results of TEER analysis and semi-quantitative WB analysis. This increase in tight junction protein synthesis might be responsible for an increased SC barrier integrity resulting in increased TEER values of KO SC cultures (Fig. 6).

## Discussion

The purpose of this study was to establish and characterize primary SC cultures of the SCCx43KO model, and to analyze differences in proliferation and tight junction expression (Carette et al. 2010; Gerber et al. 2014) based on the presence (WT) or absence (KO) of Cx43. The ultimate objective will be (after initial characterization of the primary cell culture) to eventually develop immortalized KO and WT SC lines, allowing for less animal experimenting and a vaster selection of experiments than in vivo testing.

As coinciding with previous studies from the SCCx43KO (Brehm et al. 2007; Carette et al. 2010; Gerber et al. 2014; Giese et al. 2012; Hollenbach et al. 2018; Rode et al. 2018a; Sridharan et al. 2007; Weider et al. 2011a; Weider et al. 2011b), testes of adult KO mice show SC-only tubules and seminiferous tubules with occasional GC, while the WT mice depict a normal distribution of SC and GC within the seminiferous tubules (Fig. 1). As expected, the KO mice did not express any Cx43 unlike their WT littermates, which showed a distinct localization of Cx43 at the height of the BTB in both adolescent and adult mice (Gerber et al. 2014). Claudin-11 appeared to form a more diffuse distribution pattern at the height of the BTB during adolescent ages, which in return formed distinct wavy bands at the BTB in seminiferous tubules of adult WT mice. This age-dependent distribution pattern of claudin-11 has recently been described in detail in SCCx43KO and WT mice: in WT mice, a basal shift towards the BTB could be observed during pubertal development resulting in a fine linear staining in the basal area of the seminiferous epithelium at 23 days of age similar to adult WT mice. In SCCx43KO mice, this basal shift failed to appear (Hollenbach et al. 2018). These results are similar to an occludin time study, whose distinct localization towards the BTB begins earlier, at around day 10 p.p. in WT and day 11 p.p. in KO mice (Gerber et al. 2014). Interestingly, claudin-11 KO mice also develop male sterility (Gow et al. 1999; Mazaud-Guittot et al. 2010; Morrow et al. 2010), coinciding with infertility known from adult SCCx43KO mice (Brehm et al. 2007; Sridharan et al. 2007). These results emphasize the importance of SC, Cx43 and claudin-11 in spermatogenesis, and provide an interesting aspect for testing these SC in an in vitro environment.

After the preliminary testing using testicular tissue, a proper characterization of the primary SC culture was required. No obvious morphological differences could be determined between primary KO and WT SC cultures via phase contrast microscopy, RNA expression, IHC or IF. Both KO and WT SC showed the typical morphological structure (Fig. 3a, b), spindle shaped, which has been described in numerous publications (Hofmann et al. 2003; Mather and Sato 1979; Nenicu et al. 2007).

Finally, Cx43 presence or absence in WT and KO primary SC cultures was demonstrated. As expected, the present study shows at RNA and protein level that Cx43 is present in WT primary SC culture (Fig. 4a; Fig. 7b), while the protein is absent in primary KO SC (Fig. 4b). The immunolocalization of Cx43 as small dots within the plasma membrane at contact sites between adjacent SC in the WT culture (Fig. 4a) is in accordance with previous observations (Lablack et al. 1998). Cx43 has a short half-life, so a continuous junction remodeling is often necessary and gap junction components have to be delivered constantly to the cell borders (Epifantseva and Shaw 2018). Thus, the intracellularly identified Cx43 possibly reflects different steps of protein synthesis and junction formation (endoplasmic reticulum, Golgi apparatus) (Epifantseva and Shaw 2018; Lablack et al. 1998), which might also be the case in the present study. In conclusion, the characterization via RNA expression, phase contrast microscopy, IHC and IF indicated no obvious difference between the KO and WT; both genotypes yielded an extremely pure SC culture.

After successfully characterizing pure primary KO and WT SC cultures, the proliferation rate was determined in vitro. Initially, it was hypothesized (based on in vivo data from (Brehm et al. 2007, Sridharan et al. 2007)) that KO SC might potentially proliferate at a different rate through the lack of Cx43. This theory was based on the finding that the absence of the gap junction protein caused a lack of communication between adjacent cells, in return causing the cells to potentially proliferate more and/or longer (Sridharan et al. 2007). Nevertheless, Fig. 5 indicates that there were no significant differences between the KO and WT proliferation rates in cell culture between days 4, 6, and 8 after seeding out, not ruling out yet that KO SC might be capable of prolonged/increased proliferation though not statistically significant. This finding emphasized that a simple transmission of in vivo data to in vitro systems (and vice versa) is not necessarily possible, because especially in vivo there are (maybe still unknown) additional influences (e.g., substances, other cell types, hormones), which an in vitro system might not be able to comply with.

In a microarray study, a total of 658 genes were significantly and differently regulated in testes of 8 day old SCCx43KO mice compared to their WT littermates (Giese et al. 2012). It has been well established that there is an evident correlation between testicular gap junction and tight junction expression (Carette et al. 2010; Cheng and Mruk 2012; Fink et al. 2006; Gerber et al. 2014; Giese et al. 2012; Mok et al. 2011; Segretain et al. 2004; Tripathi and Tripathi 2010). Studies from Carette et al. (2010) stated that the use of siRNA for Cx43 in a SerW3 rat SC line resulted in a significantly increased occludin protein synthesis in these cultures in comparison to those without Cx43-siRNA. Carette et al. (2010) also analyzed testes of adult SCCx43KO mice, where it was shown that KO mice synthesized significantly more occludin and less of the tight junction associated protein ZO-1. Additional IF studies indicated that these changes were particularly evident at the BTB (Carette et al. 2010). In addition, a quantitative PCR analysis of adult SCCx43KO mice revealed an increased expression in the tight junction genes occludin and claudin-11 (Gerber et al. 2014). In the same publication, IHC revealed an altered spatio-temporal expression pattern of occludin in prepubertal SCCx43KO mice. Specifically, the lack of Cx43 seemed to have caused a delay of the shift of occludin towards the BTB region at the ages of 10–12 days p.p. (Gerber et al. 2014). These publications support the results of the present TEER analysis (Fig. 6) and subsequent investigation of claudin-11 protein synthesis and localization (Figs. 8 and 9) in which Cx43-deficient SC cultures exhibit a significantly higher resistance than the WT cells due to more tight junctions. These results support that Cx43 exerts an important influence on tight junction synthesis in vitro. Emphasizing the crucial regulatory role of Cx43 in (tight) junction remodeling, Li et al. (2016) could show that an overexpression of Cx43 was able to reseal toxicant-induced BTB disruption and reinitiated meiosis (Li et al. 2016).

The present TEER results coincide with those found in literature, in which values around ~ 50–80 Ω cm^2^ from rodent SC cultures without the addition of any hormones such as follicle-stimulating hormone (FSH) or testosterone appear as a standard. It is well known that these SC cultures reach their peak resistance after 3–4 days of culture and remain constant thereafter for numerous days (Fig. 6) (Mruk and Cheng 2011). These publications also indicate significant changes in the magnitude of ~ 10–40 Ω cm^2^ after administering various substances, which alter tight junction expression (Chung and Cheng 2001; Kaitu'u-Lino et al. 2007; Li et al. 2014; Mruk and Cheng 2011; Siu et al. 2009a; Siu et al. 2009b; Zhang et al. 2008). Hence, the magnitude of change, ~ 17 Ω cm^2^, between the KO and WT seems to be an acceptable significant change and indicates the importance of Cx43 in regulating on the tight junction barrier function. Accordingly, an increased TEER value concomitant with delocalized Cx43 has been shown in vitro in response to chromium exposure (Carette et al. 2013). It is possible that the lack of communication via Cx43 based gap junction between SC during initial cell attachment causes an increased production of tight junctions during the first 4 days of culture (the initiation of cell monolayer). Furthermore, Chung et al. (1999) proposed that the initial attachment of SC within primary cell cultures was made possible by the existing adherens and gap junction molecules (Chung et al. 1999). Hence, it is possible that the increase in TEER is likely due to the absence of Cx43 in the KO cultures, in which the lack of SC-SC communication is attempted to be compensated via an over-expression of tight junction proteins during initial cell attachment. Thus, it could be proposed that inter-SC communication via Cx43 inhibits an excessive synthesis of numerous tight junctions supporting its role as a possible regulator of BTB formation, composition, and dynamics as also proposed by Gerber et al. (2016); a similar theory has also been proposed by Carette et al. (2010). However, the absence of these Cx43 gap junctions does not seem to affect the “static” maintenance and function of these tight junctions between days 4 and 8 of SC culture (Fig. 6). These results are supported by an in vitro SC Cx43 knockdown investigation, in which it was concluded that the gap junction (Cx43) is necessary to maintain the “dynamic” aspect of the BTB but not its “static” function (Li et al. 2010). Correspondingly, SCCx43KO mice are also able to form functional tight junctions and an intact BTB during pubertal development in vivo*,* which appears to be even accelerated compared with WT littermates, but the dynamic restructuring during spermatogenesis seems to be impaired (Hollenbach et al. 2018).

After TEER analysis, an evaluation of possible tight junction mRNA expression was performed (Fig. 7). The mRNA of the tight junction proteins claudin-11, occludin, and ZO-1 were detected in both KO and WT primary SC cultures. Interestingly, the KO primary cell culture still produced mRNA coding for Cx43 (a weaker band than in the WT). This can be explained by the presence of some peritubular cells within the primary cell culture as seen in Fig. 2 and Supplemental Fig. 1. The synthesis of Cx43 in rodent peritubular cells has been described by Risley et al. (1992). As described previously, claudin-11 is vital for murine BTB development and fertility as is Cx43 in the SCCx43KO model (Gow et al. 1999; Mazaud-Guittot et al. 2010; Morrow et al. 2010). Thus, the present study focused on the claudin-11 tight junction synthesis in the KO and WT SC.

A protein analysis of claudin-11 in primary SC cultures indicated a trend to a slightly increased synthesis in Cx43 deficient SC (Fig. 8a and b). Confirming the results of claudin-11 IHC (Hollenbach et al. 2018), it has been shown by semi-quantitative WB analysis in the present study that claudin-11 protein is significantly increased in adult KO mice (Fig. 8a and b), which coincides with an upregulation of *Cldn11* mRNA in the SCCx43KO adult mice (Gerber et al. 2014). Thus, in terms of claudin-11 protein synthesis, the primary SC culture model of the present study could not fully recapitulate the findings in the adult testicular tissue (Fig. 8). In addition to the observed trend that claudin-11 is elevated in KO cell culture (Fig. 8a and b, 9b and c), further analyses of other tight junction proteins (e.g., junctional adhesion molecules (JAMs) or other claudins (e.g., claudin-3 and claudin-5) may provide a greater insight into why KO mice exhibit a significantly higher electrical resistance in TEER experimentation. It is possible that different tight junction proteins are elevated in SCCx43KO cultures, which has been previously reported/supposed by Carette et al. (2010) and Gerber et al. (2014). Thus, the authors propose three theories for the upregulation of TEER: (1) a single tight junction protein is significantly upregulated; (2) a combination of multiple tight junction proteins being slightly upregulated, but not significantly; and (3) multiple tight junction proteins are significantly upregulated.

In a study investigating human testes, it was shown that seminiferous tubules of men with testicular CIS show an upregulation and dislocation of claudin-11 (Fink et al. 2009). These results have been confirmed by recent studies investigating testes of infertile men with primary seminiferous tubule failure (Chiba et al. 2012; Haverfield et al. 2013; McCabe et al. 2016). Additionally, CIS can be associated with a downregulation of Cx43 (Brehm et al. 2002; Brehm et al. 2006) and an impaired status of SC differentiation (Rajpert-De Meyts and Skakkebaek 1993; Skakkebaek et al. 1998). As claudin-11 exerts a crucial role in BTB function, its regulation was subject of various studies (Florin et al. 2005; Hellani et al. 2000; Jegou 1992; Kaitu'u-Lino et al. 2007; McCabe et al. 2016; Morrow et al. 2010). Among others (e.g., hormonal regulation via FSH or testosterone), GC seem to influence/modulate claudin-11 gene expression and protein synthesis and/or localization (Florin et al. 2005; Jegou 1992; Morrow et al. 2010; Nicholls et al. 2009) and this regulating factor is missing in single cell type culture of pure SC like in the present study. Thus, co-culturing of SC and GC is important and interesting when investigating GC-derived contributing factors on SC function. However, there are several studies using single cell type SC cultures for investigating SC junction in vitro (Bekheet and Stahlmann 2009; Chung et al. 1999; Kaitu'u-Lino et al. 2007; Lablack et al. 1998; Li et al. 2019; Mruk and Cheng 2011). Hence, the present in vitro SC model might be an ideal supplementation to determine the underlying mechanisms for altered BTB assembly and formation in human testis and further CIS investigations. However, a direct transmission of results obtained using pure SC culture models to the in vivo situation should always be taken carefully due to lack of reciprocal modulation via GC. Future studies using the herein described Cx43 KO SC culture in co-culture with GC could offer important insights about the role of Cx43 in the regulatory influence of GC on SC barrier function.

Another interesting aspect for this SC culture model can be male contraception. With two cell lines from this model, it would be possible to test potential drugs, which can then either supress Cx43 or block Cx43 communication between two SC and between SC and GC reversibly. These drugs might then act as a “switcher” in turning on or off spermatogenesis via Cx43 that is known to be a potential regulator of tight junctions in SC (Carette et al. 2010; Cyr et al. 1999; Pelletier 1995; Segretain et al. 2004).

Nevertheless, before establishing a permanent (and immortalized) cell line using cells from day 4 of culture, when functional tight junctions seemed to have formed, the primary SC cultures need to be further characterized. In follow-up studies, it is the goal to determine additional differences between KO and WT primary cell cultures through use of, e.g., PCR-bioarrays, microarrays, or next generation sequencing (NGS). Furthermore, co-culture with GC might offer insights about the possible function of Cx43 in the regulatory role of GC on SC function. Data may then provide additional information on molecular pathways influencing, e.g., tight junction expression in the presence/absence of Cx43, and provide a better understanding of the role of Cx43 within the seminiferous epithelium. Additional tight junction molecules like JAM-A, claudin-3 and claudin-5 will be analyzed through semi-quantitative WB, qRT-PCR and IHC.

In summary, using a modified 3-step enzymatic digestion, it was possible to achieve two > 98% pure primary SC cultures. Apart from Cx43 expression, no obvious morphological differences between the KO and WT cells could be determined; however, shown for the first time, an increase of tight junction protein claudin-11 was detectable in testes of adult KO mice. Even though no significant differences in claudin-11 protein in the primary cell cultures could be determined using WB, IF analysis still indicates a slight increase of claudin-11 in Cx43 deficient SC, which is supported by the present TEER results. The TEER increase discovered in the KO culture is likely due to the observation that the lacking SC-SC communication via the Cx43-based gap junction channels caused an overexpression of various tight junction proteins including claudin-11 during initial cell attachment of the primary cell culture. Thus, it could be proposed that SC-SC communication via Cx43 seemed to inhibit the synthesis of an excessive amount of tight junctions. Next steps would be to further characterize this primary SC culture by investigating additional days of culture and by analyzing gene expression using NGS and subsequently to transfect the cells to obtain an immortalized Cx43 KO SC line. It is finally our objective to provide the scientific community with beneficial SC lines (with and without Cx43), to perform less animal experiments and to develop mechanistic hypotheses of how Cx43 in SC regulates tight junction expression, BTB formation, SC maturation and ultimately spermatogenesis. These new cell lines would be an ideal model to study male factor infertility due to impaired BTB formation and to discover new possible mechanistic pathways for male contraceptives via the target molecule Cx43.

## Electronic supplementary material

ESM 1 A video of two representative histological tissue sections of an 18 day old post partum knockout (KO) mouse testis is shown. The first image is a hematoxylin eosin (HE) staining and the second image is an overlay of the consecutive tissue section stained for vimentin. These fade back and forth a couple times. The arrow indicates a fixation point which was used to align the images; while the box represents a mitotic figure in the HE section and the intermediate filament, vimentin, stained Sertoli cell of the consecutive section (MPEG 3222 kb)

ESM 2 A video of two representative histological tissue sections of a 19 day old post partum wild type (WT) mouse testis is shown. The first image is a hematoxylin eosin (HE) staining and the second image is an overlay of the consecutive tissue section stained for vimentin. These fade back and forth a couple times. The arrows indicate fixation points which were used to align the images; while the box represents a mitotic figure in the HE section and the intermediate filament, vimentin, stained Sertoli cell of the consecutive section (MPEG 3222 kb)

ESM 3 A video of two representative histological tissue sections of an 18 day old postnatum knockout (KO) mouse testis is shown. The first image is a hematoxylin (HE) staining and the second image is an overlay of the consecutive tissue section stained for Sox9. These fade back and forth a couple times. The arrow indicates a fixation point which was used to align the images; while the boxes represent mitotic fugures in the HE section and Sox9 nuclear stained Sertoli cell of the consecutive section (MPEG 3222 kb)

ESM 4 A video of two representative histological tissue sections of a 19 day old post partum wild type (WT) mouse testis is shown. The first image is a hematoxylin (HE) staining and the second image is an overlay of the consecutive tissue section stained for Sox9. These fade back and forth a couple times. The arrows indicate fixation points which were used to align the images; while the boxes represent mitotic figures in the HE section and Sox9 nuclear stained Sertoli cell of the consecutive section (MPEG 3222 kb)

ESM 5 Representative immunolocalization of smooth muscle actin (SMA) in primary Sertoli cell (SC) culture. SMA depicts few remaining peritubular cells in the primary SC cultures (image a: magnification x100; image b: magnification x200). It is visible that the SC culture is highly pure and no visual differences could be determined between knockout and wild type (WT) during the staining process. Images stem from representative WT SC cultures (PNG 607 kb)

High Resolution Image (TIF 4427 kb)
