# Supplementary figures and images for: Establishment and functional characterization of a murine primary Sertoli cell line deficient of connexin43
Source: Cell Tissue Res. 2020 Apr 23;381(2):309–26. doi: 10.1007/s00441-020-03203-y (PMC7369266; doi:10.1007/s00441-020-03203-y)

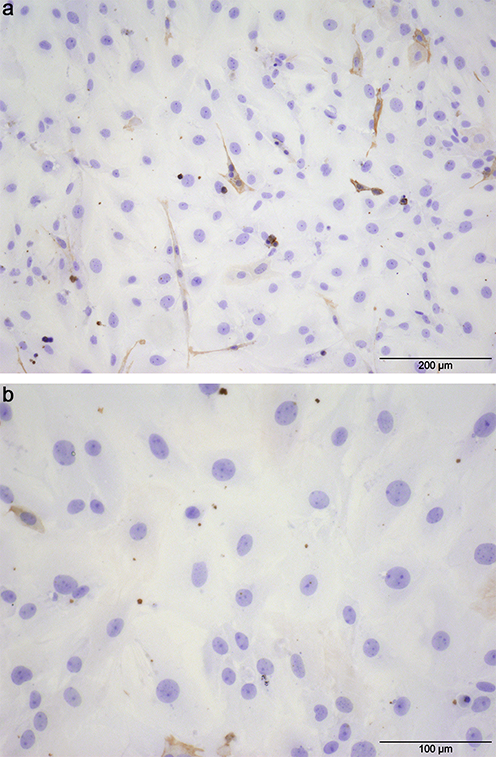

Supplement: Supplementary file 5 — Representative immunolocalization of smooth muscle actin (SMA) in primary Sertoli cell (SC) culture. SMA depicts few remaining peritubular cells in the primary SC cultures (image a: magnification x100; image b: magnification x200). It is visible that the SC culture is highly pure and no visual differences could be determined between knockout and wild type (WT) during the staining process. Images stem from representative WT SC cultures (PNG 607 kb) [file 441_2020_3203_Fig10_ESM.png]

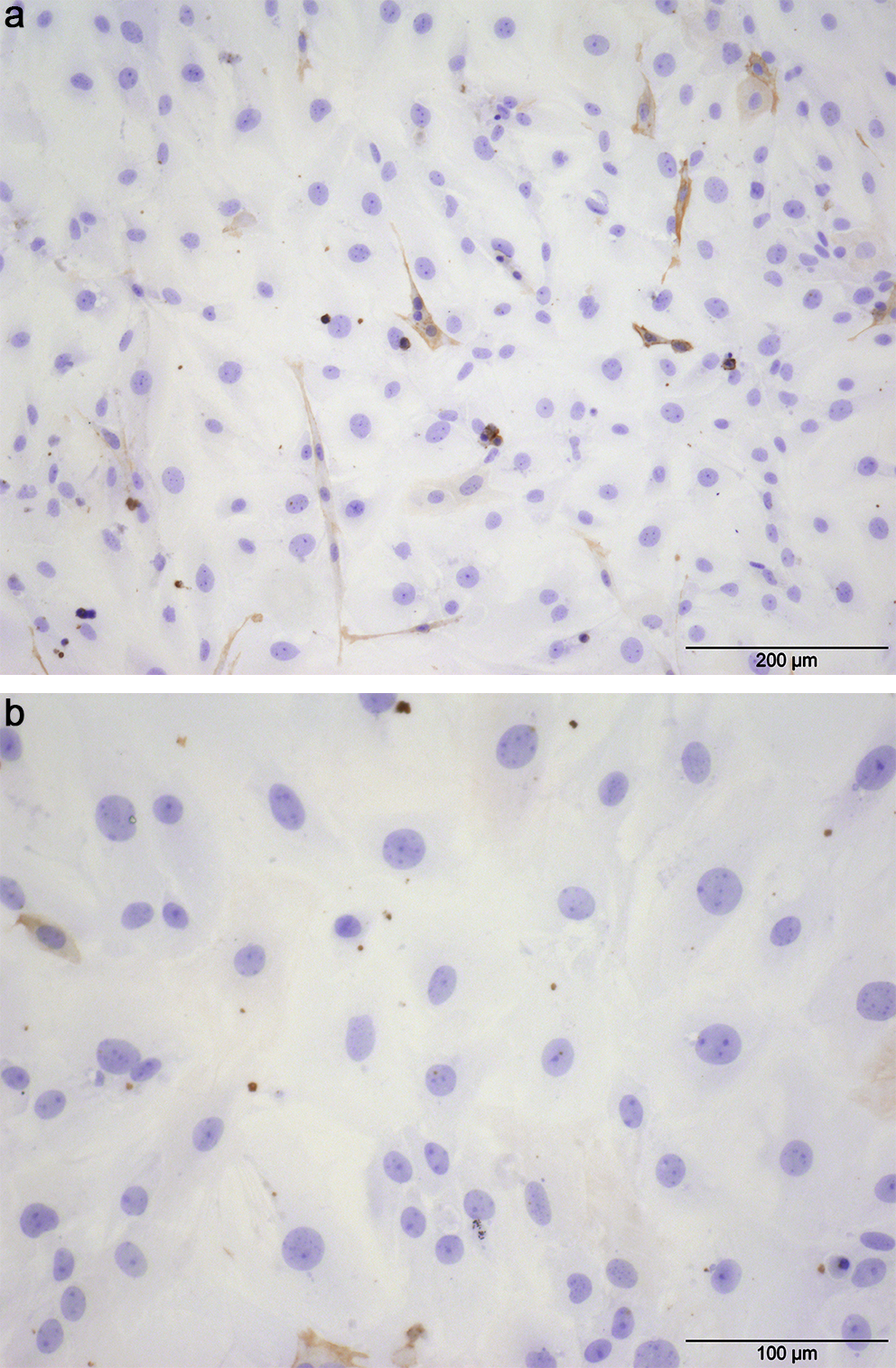

Supplement: Supplementary file 6 — High Resolution Image (TIF 4427 kb) [file 441_2020_3203_MOESM5_ESM.tif]
